# Expressing gK gene of duck enteritis virus guided by bioinformatics and its applied prospect in diagnosis

**DOI:** 10.1186/1743-422X-7-168

**Published:** 2010-07-21

**Authors:** Shunchuan Zhang, Guangpeng Ma, Jun Xiang, Anchun Cheng, Mingshu Wang, Dekang Zhu, Renyong Jia, Qihui Luo, Zhengli Chen, Xiaoyue Chen

**Affiliations:** 1 Avian Disease Research Center, College of Veterinary Medicine of Sichuan Agricultural University, 46# Xinkang Road, Ya'an, Sichuan 625014, China; 2 China Rural Technology Development Center, Beijing, 100045, China; 3 Key Laboratory of Animal Disease and Human Health of Sichuan Province, Ya'an 625014, China; 4 Epizootic Diseases Institute of Sichuan Agricultural University, Ya'an, Sichuan 625014, China

## Abstract

**Background:**

Duck viral enteritis, which is caused by duck enteritis virus (DEV), causes significant economic losses in domestic and wild waterfowls because of the high mortality and low egg production rates. With the purpose of eliminating this disease and decreasing economic loss in the commercial duck industry, researching on glycoprotein K (gK) of DEV may be a new kind of method for preventing and curing this disease. Because glycoproteins project from the virus envelope as spikes and are directly involved in the host immune system and elicitation of the host immune responses, and also play an important role in mediating infection of target cells, the entry into cell for free virus and the maturation or egress of virus. The gK is one of the major envelope glycoproteins of DEV. However, little information correlated with gK is known, such as antigenic and functional characterization.

**Results:**

Bioinformatic predictions revealed that the expression of the full-length gK gene (*fgK*) in a prokaryotic system is difficult because of the presence of suboptimal exon and transmembrane domains at the C-terminal. In this study, we found that the *fgK *gene might not be expressed in a prokaryotic system in accordance with the bioinformatic predictions. Further, we successfully used bioinformatics tools to guide the prokaryotic expression of the *gK *gene by designing a novel truncated *gK *gene (*tgK*). These findings indicated that bioinformatics provides theoretical data for target gene expression and saves time for our research. The recombinant tgK protein (tgK) was expressed and purified by immobilized metal affinity chromatography (IMAC). Western blotting and indirect enzyme-linked immunosorbent assay (ELISA) showed that the tgK possessed antigenic characteristics similar to native DEV-gK.

**Conclusions:**

In this work, the DEV-*tgK *was expressed successfully in prokaryotic system for the first time, which will provide usefull information for prokaryotic expression of alphaherpesvirus gK homologs, and the recombinant truncated gK possessed antigenic characteristics similar to native DEV gK. Because of the good reactionogenicity, specificity and sensitivity, the purified tgK could be useful for developing a sensitive serum diagnostic kit to monitor DEV outbreaks.

## Background

Duck viral enteritis is caused by the duck enteritis virus (DEV). DEV has been included in the subfamily *Alphaherpesvirinae *of the family *Herpesviridae*, but it has not been grouped into a genus [1]. DEV has an icosahedral capsid containing a double-stranded linear DNA with 64.3% G + C content, which is higher than that of any other reported avian herpesvirus in the subfamily *Alphaherpesvirinae *[2]. The nucleocapsid is surrounded by a tegument, which is enclosed by an envelope with integral viral glycoproteins [3].

DEV causes an acute, contagious, and highly lethal disease in birds of all ages from the order Anseriformes (ducks, geese, and swans) [4-7]. The disease is characterized by vascular damage, tissue hemorrhage, digestive mucosal eruptions, lesions of lymphoid organs, and degenerative changes in parenchymatous organs [8,9]. Reactivation of latent virus has the possibility of causing outbreaks of duck viral enteritis in domestic and migrating waterfowl populations [10]. In duck rearing areas of the world where the disease has been reported, duck viral enteritis has caused significant economic losses because of the high mortality and low egg production rates [11,12].

With the purpose of eliminating this disease and decreasing economic losses in the commercial duck industry, studying glycoprotein K (gK) of DEV may be a new method for preventing and curing this disease. Glycoproteins are the major antigens recognized by the infected host's immune system and play an important role in mediating target cell infection, cellular entry of free viruses, and the maturation or egress of the virus [13,14]. Glycoprotein K is one of the major glycoproteins encoded by the *DEV-gK *gene, which is located in the unique long region of the DEV genome. Additionally, gK is capable of inducing a protective immune response in vivo and is responsible for viral binding to the cellular receptor [15].

To date, some genes from the DEV genome have been identified, but little is known about the *gK *gene [16-23]. The objective of this study was to report on *DEV-gK *gene expression, as guided by bioinformatics, and to purify DEV-gK and analyze its immunoreactivity. The findings will provide some insights for further study of the gene and will lead to the development of new strategies for preventing this disease.

## Results

### Design of *tgK *as guided by bioinformatics software and web service

**Figure 1:**
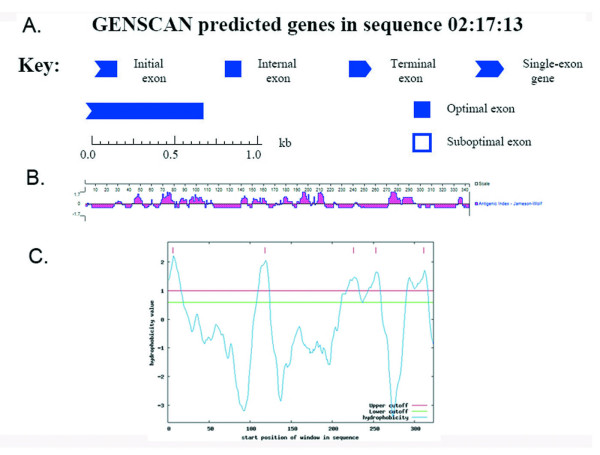
**The *fgK *and its full-length amino acid sequence were analyzed by on-line prediction tools and DNASTAR7.0 software**. **A **represented that the optimal exon domain was predicted by GENSCAN http://genes.mit.edu/GENSCAN.html. **B **represented that the potential antigenic epitopes of the gK was analyzed by DNASTAR7.0 software. **C **represented hydrophilicity domains of the gK was predicted on line http://mobyle.pasteur.fr/cgi-bin/portal.py?form=toppred.

The GENESCAN prediction online indicated that the integral ORF of the *DEV-gK *gene was divided into 2 parts, which contained an optimal exon domain from 1 to 675 bp and a suboptimal exon domain from 676 to 1032 bp. In addition, the corresponding 225 aa polypeptide chain, encoded by the optimal exon domain shown in blue in Fig. 1A, could be easily expressed, however, the suboptimal exon domain might be hard to express according to the predicted result. To estimate the potential epitopes of DEV-gK, the amino acid sequence of DEV-gK was analyzed using DNASTAR 7.0 software. The putative DEV-gK epitopes thus identified were mainly located from amino acids 25-115, 135-215, and 270-295, with corresponding DNA sequences at nucleotides 73-345, 403-645, and 808-885 (Fig. 1B). Hydrophilicity estimation and transmembrane region assumption were performed using online prediction tools. Hydrophilic domains were mainly located from amino acids 7-27, 119-139, 227-247, 254-274, and 312-332 (Fig. 1C). Moreover, transmembrane regions were identified in 5 amino acid stretches, 7-29, 118-140, 220-242, 252-274, and 313-335. From the above data, the hydrophilic domains and transmembrane regions exist in a one-to-one ratio. To design the *tgK *gene (91-642 bp), the 4 predicted results were combined together and included the gK amino acids 31-214. Additionally, the tgK possessed good immunogenicity, determined by prediction, and only one potential transmembrane region. These results suggested that expression of tgK might be possible.

### The construction and sequencing of cloning plasmid

**Figure 2:**
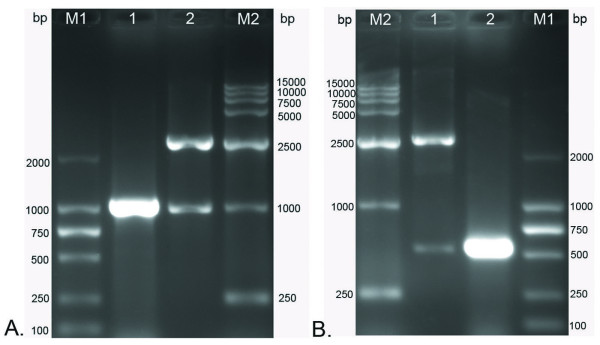
**The construction of cloning plasmids were identified by colony PCR and restriction enzymes (*Hind*III and *Xho*I) digestion**. M1, DNA marker-DL2000; M2, DNA marker-DL15000. **A**. Identification of cloning plasmid pMD18-T/*fgK*. Lane 1, product of the colony PCR; Lane 2, two fragments after digestion of the recombinant DNA with restriction enzymes *Hind*III and *Xho*I. **B**. Identification of cloning plasmid pMD18-T/*tgK*. Lane 1, two fragments after digestion of the recombinant DNA with restriction enzymes *Hind*III and *Xho*I; Lane 2, product of the colony PCR.

By using high fidelity Taq enzyme to isolate the *gK *gene, PCR was carried out on DNA from the DEV genome. *fgK *was amplified by one pair of primers (P1, P2) and *tgK *was amplified by another primer set (P3, P4). Both pairs of primers were specific to the *DEV-gK *gene, seen through the screening for expected products (Fig. 2). The *fgK *PCR product was approximately 1000 bp and that of *tgK *was approximately 550 bp, which were respectively amplified and cloned into pMD18-T and were identified through colony PCR and restriction enzymes (*Hin*dIII and *Xho*I) digestion. Thus, positive recombinant plasmids was constructed for *fgK *and *tgK*, and were designated as pMD18-T/*fgK *(Fig. 2A) and pMD18-T/*tgK *(Fig. 2B), respectively. The sequencing results of both cloning plasmids showed that there were no nucleotide errors in the synthetic *fgK *gene and *tgK *gene fragment (data not shown).

### The construction of expression plasmid and sequencing

**Figure 3:**
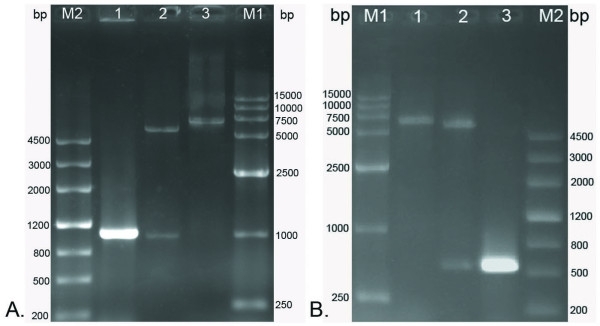
**The construction of expression plasmids were identified by colony PCR, single restriction enzyme *Hind*III digestion and two restriction enzymes (*Hind*III and *Xho*I) digestion**. M1, DNA marker-DL15000; M2, DNA MarkerIII. A. Identification of the expression plasmid pET-32b(+)/*fgK*. Lane 1, the colony PCR; Lane 2, two fragments after digesting the recombinant DNA with restriction enzymes *Hind*III and *Xho*I; Lane 3, one band by single restriction enzyme *Hind*III digestion. **B**. Identification of the expression plasmid pET-32b(+)/*tgK*. Lane 1, one band by single restriction enzyme *Hind*III digestion; Lane 2, two fragments after digesting the recombinant DNA with restriction enzymes *Hind*III and *Xho*I; Lane 3, the colony PCR.

The prokaryotic expression plasmid pET-32b(+), which possesses a high stringency T7 lac promoter, His tag, and T7 terminator, has been recognized as one of the most powerful tools for producing recombinant proteins in *Escherichia coli*. The *fgK *and *tgK *fragments, which were respectively obtained by digestion of pMD18-T/*fgK *and pMD18-T/*tgK *with *Hin*dIII and *Xho*I, were directionally inserted into the pET-32b(+) plasmid to construct the expression plasmids pET-32b(+)/*fgK *and pET-32b(+)/*tgK*, respectively. The initial expression plasmids were transformed into competent *E. coli *DH5α cells for the purpose of screening. The expression plasmids were identified by PCR, 2 restriction enzymes digestion (*Hin*dIII and *Xho*I), and single restriction enzyme digestion with *Hin*dIII (Fig. 3). As shown in Fig. 3A, an approximately 1000 bp band was amplified by PCR, band approximately 1000 bp and 5900 bp were observed following 2 restriction enzymes digestion, and an approximate 6000 bp band was observed by single restriction enzyme digestion, which corresponded to *fgK *(1032 bp) and the pET-32b(+) plasmid (5900 bp), respectively. As shown in Fig. 3B, for *tgK*, PCR amplified a band approximately 500 bp, the bands produced from digestion using 2 restriction enzymes were 500 bp and 5900 bp, and the band resulting from single restriction enzyme digestion was about 6000 bp, all of which corresponded to *tgK *(552 bp) and the pET-32b(+) plasmid (5900 bp), respectively. Subsequently, positive pET-32b(+)/*fgK *and pET-32b(+)/*tgK *clones were submitted for DNA sequencing, thereby confirming that the *fgK *and *tgK *genes were respectively inserted into the pET-32b(+) multiple cloning site (data not shown).

### Expression of the recombinant protein and optimization of expressing conditions

**Figure 4:**
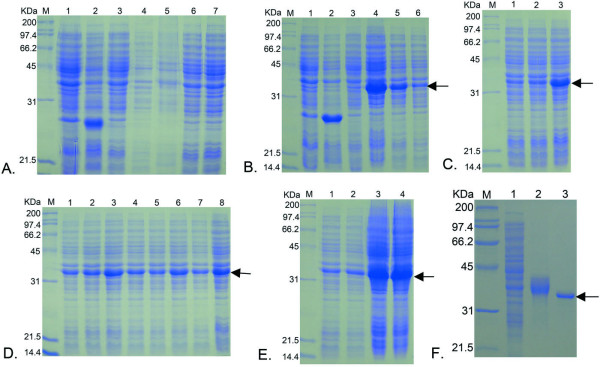
**The different samples were analyzed by Coomassie blue-stained polyacrylamide gel.** The direction of arrow represented the target protein. M represented standard protein molecular weight markers. **A**. Lane 1, the un-induced BL21 bacteria within pET-32b(+) plasmid; Lane 2, the induced BL21 bacteria within pET-32b(+) plasmid; Lane 3, the un-induced BL21 bacteria within pET-32b(+)/*fgK *plasmid; Lane 4, the supernatant of IB with sonication was analyzed by SDS-PAGE, that the IB was harvested from the induced BL21 bacteria within pET-32b(+)/*fgK *plasmid; Lane 5, the pellet of IB with sonication was analyzed by SDS-PAGE, that the IB was harvested from the induced BL21 bacteria within pET-32b(+)/*fgK *plasmid; Lane 6, the induced Plys bacteria within pET-32b(+)/*fgK *plasmid; Lane 7, the induced Rosetta bacteria within pET-32b(+)/*fgK *plasmid. **B**. Lane 1, the un-induced BL21 bacteria within pET-32b(+) plasmid; Lane 2, the induced BL21 bacteria within pET-32b(+) plasmid; Lane 3, the un-induced BL21 bacteria within pET-32b(+)/*tgK *plasmid; Lane 4, the induced BL21 bacteria within pET-32b(+)/*tgK *plasmid; Lane 5, the induced Plys bacteria within pET-32b(+)/*tgK *plasmid; Lane 6, the induced Rosetta bacteria within pET-32b(+)/*tgK *plasmid. **C**. The determination of the optimal induced temperature. Lanes 1, 2 and 3 representing the induced temperatures were 25°C, 30°C and 37°C, respectively. **D**. The determination of the optimal induced IPTG final concentration. Lanes 1, 2, 3, 4, 5, 6, 7 and 8 representing the induced IPTG final concentrations were 0.2 mM, 0.3 mM, 0.4 mM, 0.5 mM, 0.6 mM, 0.7 mM, 0.8 mM and 1.0 mM, respectively. **E**. The determination of the optimal induced duration. Lanes 1, 2, 3 and 4 representing the induced durations were 2.0 h, 4.0 h, 8.0 h and 16.0 h, respectively. **F**. Purification of IB and recombinant truncated gK. Lanes 1 and 2 respectively represented the supernatant and pellet of the induced bacteria within pET-32b(+)/*tgK *plasmid with sonication; Lane 3 represented the purified recombinant truncated gK by a single chromatographic step of IMAC on Ni^2+^-NTA agarose.

After sequence confirmation, the recombinant expression plasmids pET-32b(+)/*fgK *and pET-32b(+)/*tgK *were transformed into the expression host *E. coli *BL21. The positive transformants were selected for recombinant protein expression. Initially, the expression of fgK and tgK were induced at 37°C for 4 h by the addition of 0.2 mM IPTG. The induced pET-32b(+)/*fgK *was compared with uninduced pET-32b(+)/*fgK *culture and induced and uninduced bacteria carrying an empty pET32b(+) vector by SDS-PAGE. However, a band with a molecular weight approximately 57 kDa was not observed (Fig. 4A, lanes 4 and 5). Therefore, the recombinant pET-32b(+)/*fgK *expression plasmid was transformed into other expression hosts (Plys, Rosetta) with the same result.

The recombinant pET-32b(+)/*tgK *expression plasmid was transformed into the expression host *E. coli *BL21, which was induced as described in the methods section. We compared the induction of *E. coli *BL21 pET-32b(+)/*tgK *with those that were not induced and with induced and uninduced bacteria carrying an empty pET32b(+) plasmid by SDS-PAGE, and detected a specific band of approximately 34 kDa, which was smaller than the predicted weight (tgK = 20 kDa, His-tag = 20 kDa, His-tag-tgK ≈ 40 kDa), for the induced pET-32b(+)/*tgK *culture (Fig. 4B, lane 4). This target band was not detected in the uninduced pET-32b(+)/*tgK *culture (Fig. 4A, lane 3), nor was it found in induced and uninduced bacteria carrying the pET-32b(+) plasmid (Fig. 4A, lanes 1 and 2).

The recombinant pET-32b(+)/*tgK *expression plasmid was also transformed into the expression hosts Plys and Rosetta. Using the same expression procedures for tgK, recombinant BL21 bacteria produced the higher quantities of the fusion protein tgK by about 10% in total (data not shown), which was compared with that of expression host Plys and Rosetta (Fig. 4B, lanes 4, 5, and 6).

The optimization of expression conditions as described in the methods section concerned the temperature for induction, final IPTG concentrations, and duration of induction. As in Fig. 4C, the optimal induction temperature was 37°C (Fig. 4C, lane 3) since the expression level was higher than that at 25°C (Fig. 4C, lane 1) and 30°C (Fig. 4C, lane 2). Shown in Fig. 4D, the optimal concentration for IPTG induction was 0.4 mM (Fig. 4D, lane 3), as compared with other IPTG concentrations. The optimal induction time was 8.0 h and 16.0 h (Fig. 4E, lanes 3 and 4) because the quantity of expression was higher than that at 2.0 h (Fig. 4E, lane 1) and 4.0 h (Fig. 4E, lane 2). Therefore, the optimal expression conditions for tgK were growth at 37°C for 8.0 h with 0.4 mM IPTG.

### Purification of the recombinant protein

Generally, high expression levels of recombinant proteins in *E. coli *often results in the formation inclusion bodies (IB), insoluble and inactive protein aggregates [24,25]. In this study, host bacteria transformed with pET-32b(+)/*tgK *plasmid were cultured in 2 L LB medium supplemented with ampicillin (100 μg/ml). After host bacteria collection, the distribution of the recombinant protein in the soluble supernatant or insoluble pellet was examined by sonication. The expressed recombinant protein was predominantly detected in the insoluble pellet (Fig. 4F, lane 2). This result also indicated that little soluble protein was formed (Fig. 4F, lane 1). Purification of the IB (approach described in methods) was very effective as there were few other hetero-bands detected by SDS-PAGE (Fig. 4F, lane 2).

In order to obtain a highly purified recombinant tgK product, the purified IB were solubilized as described in the methods section, and were also subjected to His-tag purification by a single immobilized metal affinity chromatography (IMAC) chromatographic step on Ni^2+^-NTA agarose. The purity of the eluted recombinant protein was analyzed by SDS-PAGE, which detected a single band on the SDS-PAGE gel following coomassie blue staining (Fig. 4F, lane 3).

### Western blot analysis

**Figure 5:**
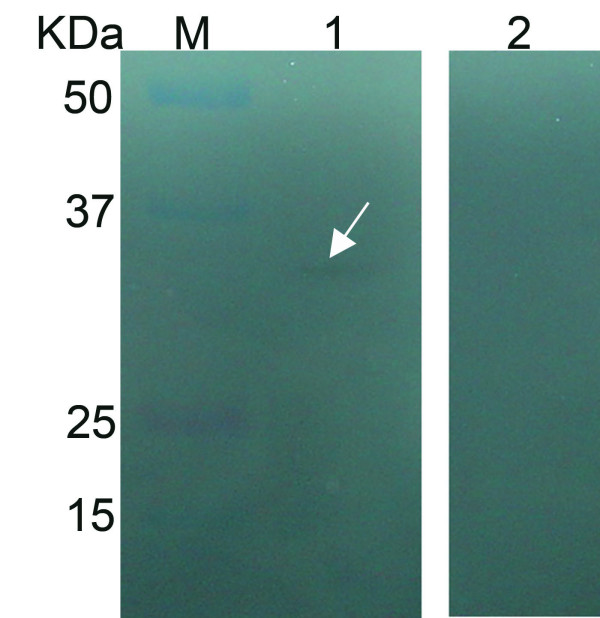
**Western blot analysis of purified truncated gK.** The direction of arrow represented the target protein. M represented bicolor prestained protein marker; Lane 1, Western blotting analysis with rabbit anti-DEV monoclonal antibody; Lane 2, Western blot analysis with negative rabbit serum.

Purified tgK, which reacted with rabbit anti-DEV polyclonal antibody, was apparent on western blots (Fig. 5, lane 1) as a single specific band approximately 34 kDa. Its purity was estimated to be greater than 95%. Meanwhile, the negative control rabbit serum did not show any reaction with tgK in western blots (Fig. 5, lane 2).

### Indirect enzyme-linked immunosorbent assay (ELISA)

To quantify the reactionogenicity, specificity, and sensitivity of tgK, an indirect ELISA assay was performed on the tgK immunized with sera samples. The optimal dilution of tgK and serum was 4 μg/ml and 1:640, respectively (shown in Table 1). To establish the cutoff value for the indirect ELISA, 32 sera samples from uninfected ducks were analyzed. The mean value of the OD_450_ for these samples, as detected by the indirect ELISA, was 0.5834, with a standard deviation of 0.1423. For a 99% confidence interval, the cutoff value was defined as follows: mean value of the OD_450_ for 32 negative sera + 3 standard deviations = 0.5834 + 3 × 0.1423 = 1.0103.

**Table 1:** The optimal dilutions of the truncated gK and sera was determined by P/N*

Dilutions of duck anti-DEV positive antiserum and duck negative serum	The dilutions of the truncated gK (μg/ml)							
	
	2	3	4	5	6	7	8	10
1:10	1.803	1.648	1.853	1.932	2.119	1.921	1.901	1.938
1:20	1.962	1.776	2.036	2.143	2.072	2.230	2.174	2.108
1:40	2.038	1.937	2.253	2.203	2.436	2.455	2.389	2.551
1:80	1.991	2.078	2.573	2.578	2.236	2.658	2.510	2.618
1:160	1.886	2.040	2.613	2.660	2.581	2.737	2.885	2.542
1:320	1.676	2.116	2.910	2.543	2.720	2.781	2.878	2.767
1:640	1.581	2.149	**3.046**	2.201	2.680	2.699	2.690	2.440
1:1280	1.552	1.970	2.760	2.236	2.424	2.748	2.723	2.874
1:2560	1.491	1.808	2.537	1.981	1.949	2.418	2.524	2.542
1:5120	1.163	1.851	1.943	1.710	1.810	2.148	2.401	2.534

*P/N represents OD_450_ (Duck anti-DEV positive serum)/OD_450_ (Duck negative serum); the dilutions of the truncated gK was 2, 3, 4, 5, 6, 7, 8 and 10 μg/ml, the result of the repeated test about the optimal dilution was same with the above.

**Figure 6:**
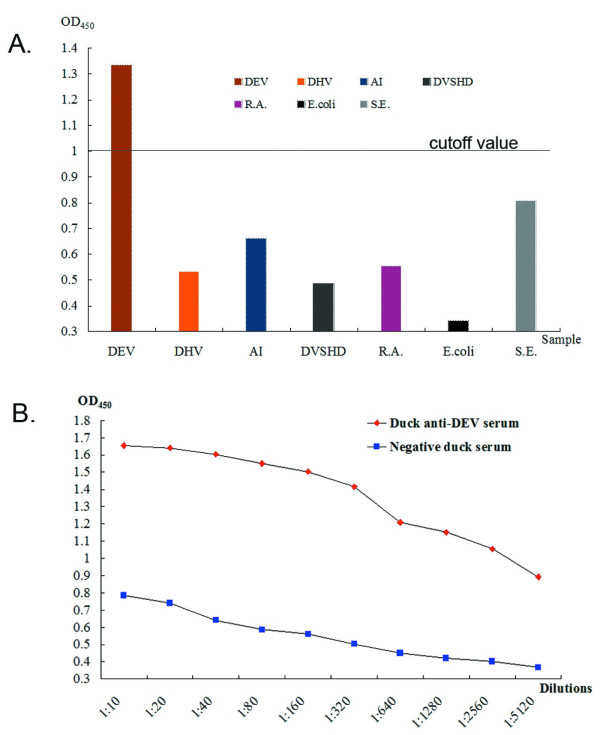
**Specificity and sensitivity of the indirect ELISA**. **A**. Specificity of the indirect ELISA. The different sera samples of duck anti-DEV, duck anti-DHV, duck anti-AI, duck anti-DVSHD, duck anti-R.A., duck anti-E. coli and duck anti-S.E. were tested by the indirect ELISA. Columns represent the mean absorbance from triplicate wells. All OD_450_ values except for DEV were lower than the cutoff value (1.0103). **B**. Sensitivity of the indirect ELISA. Different concentrations of the serum sample (from 1:10 dilution to 1:5120 dilution). At last the minimum detection limit of the duck anti-DEV positive sera was 1:2560, which could be detected by the indirect ELISA.

On the basis of the cutoff value, 7 kinds of positive sera from anti-DEV, anti-DHV (Duck hepatitis virus), anti-AI (Avian influenza, H_5_), anti-DVSHD (Duck viral swollenhead haemorrhagic disease), anti-R.A. (Riemerella anatipestifer, serotype 1), anti-E.coli (Escherichia coli, O_1_) and anti-S.E. (Salmonella Enteritidis) ducks were tested by indirect ELISA to evaluate specificity. However, except for DEV, the other sera did not yield positive results (Fig. 6A). This indicated that false-positive results were not obtained by indirect ELISA of other sera.

The sensitivity of the indirect ELISA was determined by using different dilutions of duck anti-DEV positive sera. A minimum detection limit of the duck anti-DEV positive sera was 1:2560 (OD_450_ = 1.055) according to the cutoff value (1.0103), while the negative control duck serum did not yield positive results (Fig. 6B). The detecting results of the indirect ELISA indicated that recombinant tgK reacted with duck anti-DEV positive serum in a dose-dependent manner. The indirect ELISA also had good repeatability through the course of our experiments.

## Discussion

Novel subunit vaccine strategies against herpesviruses have been based on the glycoproteins that make up the major envelope of the virus because of their location in the virion envelope and on the surface of infected cells, thereby making them important targets for the host immune system [26]. These glycoproteins act on the viral entry process in permissive cells and play important roles in pathogenicity by mediating cell-to-cell spread of the virus [27,28]. Therefore, glycoproteins are the important antigens for rapid viral diagnosis. However, to our knowledge, the immunological potential of DEV-gK has not been studied previously. Thus, the aim of this study was to express DEV-gK in a prokaryotic expression system and to purify a recombinant gK protein in order to evaluate its antigenicity and reactionogenicity.

With the progress in bioinformatics technologies, genome-wide analysis is becoming available to a broad range of research fields, such as DNA sequencing, gene and protein expression analysis, protein structure and interaction analysis, and pathway or network analysis [29]. Therefore, bioinformatics software and online analysis were applied to predict the optimal exon domains of the nucleotide sequence, potential antigenic epitopes, hydrophilic domains, and transmembrane regions of the gK amino acid sequence before carrying out the experiment. To obtain the 4 predicted results, the tgK, which possesses only 1 transmembrane region and is located in the main antigen domain, was designed and used as a candidate in lieu of fgK Therefore, these predicted results theoretically provided some useful data to guide the expression and prepare the polyclonal antibody against the recombinant protein. However, these results were just predictions that required further investigation. Our experiments showed that fgK was not expressed in *E. coli *BL21, Plys, and Rosetta as predicted, whereas tgK was better expressed in *E. coli *BL21. The lack of fgK expression might arise from the 3' noncoding region and transmembrane domains.

The *tgK *gene was PCR amplified from the DEV CHv-strain genome. The *tgK *gene was subcloned and the recombinant protein was expressed as well as purified from *E. coli *BL21. The optimal growth condition for expression of tgK was 37°C for 8.0 h with 0.4 mM IPTG. The level of purification of tgK was determined as the detection of a single band by SDS-PAGE. The identified band had a smaller molecular weight than the predicted weight of 40 kDa. This may be the result of N-glycosylation sites in tgK, a post-translational modification that could not occur in *E. coli *[30]. This phenomenon was reported in the *gK *gene of herpes simplex virus as well [31].

Western blot analysis showed that the purified His-tagged tgK was recognized by rabbit anti-DEV IgG with a signal specific band at 34 kDa. Significantly, no positive signal band was observed using the negative control serum. This result indicates that the recombinant tgK is one of the DEV glycoproteins that is generated in an immunological reaction and that the recombinant tgK had a high level of specificity to the rabbit anti-DEV IgG. Although other immunoassays were used, ELISA was one of the most sensitive and extensively applied methods to evaluate the expressed proteins. The result of the indirect ELISAs revealed that tgK possessed good reactionogenicity, specificity, and sensitivity, which could be applied in serum detection of ducks infected with DEV. Therefore, tgK has the potential to be developed as a diagnostic reagent for DEV.

## Conclusions

In summary, we successfully expressed DEV-*tgK *in a prokaryotic expression system for the first time and found that the recombinant tgK possessed antigenic characteristics similar to that of native DEV-gK by using western blotting and indirect ELISA. Because of improved reactionogenicity, specificity, and sensitivity, the purified tgK could be useful for developing a sensitive serum diagnostic kit to monitor DEV outbreaks.

## Methods

### Viruses, DEF cells and DNA template

DEV-CHv strain was a high-virulence field strain isolated in China. Duck embryo fibroblasts (DEF) were cultured in MEM medium supplemented with 10% fetal bovine serum (FBS) at 37°C [18,30]. For virus infection, MEM medium supplemented with 2-3% FBS was used. When 80%-90% of cells showed cytopathology in the form of apparent vacuoles, viruses were harvested. DEV from DEF were harvested by three freeze-thaw cycles and clarified by centrifuging for 10 min at 10, 000 g in a F2402H rotor (Beckman,USA). DEV viral DNA was extracted as described by Hansen et al [32].

### Designing truncated gK gene (*tgK*) guided by bioinformatics software and web service

Considering the uncertainty of prokaryotic system is able to express fgK, therefore bioinformatics software and web service are applied to analyze the optimal exon of gK gene, and analyze the antigenic determinants, hydrophilicity as well as transmembrane region of the gK. The structure of optimal exon was analyzed by using Genscan http://genes.mit.edu/GENSCAN.html[33]. The DNAStar 7.0 gave us a pathway to predict the antigenicity. The website located at http://mobyle.pasteur.fr/cgi-bin/portal.py?form=toppred gave us the hydrophilicity prediction of the gK. A transmembrane region analysis was executed by http://www.cbs.dtu.dk/services/TMHMM-2.0/. Combining the four analyses we designed tgK, which possessed good immunogenicity and at least could be expressed in theory.

### Construction of the cloning plasmid and sequencing

**Figure 7:**
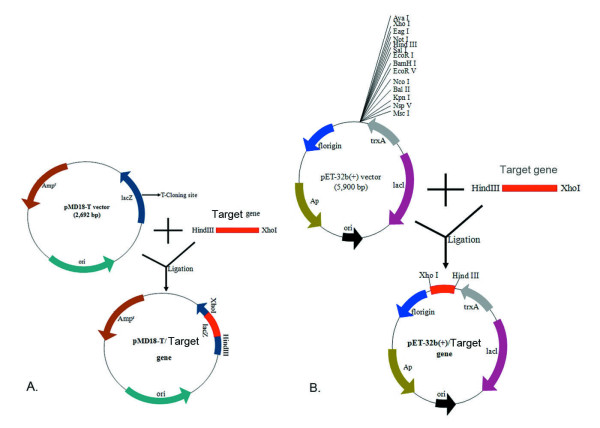
**Schematic diagram of the target gene cloned into the pMD18-T cloning plasmid and subcloned into the pET-32b(+) expression plasmid**. **A**. Schematic diagram of the target gene cloned into the pMD18-T cloning plasmid. **B**. Schematic diagram of the target gene cloned into the pET-32b(+) expression plasmid.

The full-length gK gene (*fgK*) of DEV [GenBank accession no. EU071035] was amplified with one pair of primers. Forward primer (P1) 5'-**AAGCTT**CCGCCAATAATGTTCTTAGG -3' and the reverse primer (P2) 5'-**CTCGAG**GCAAATTTATGCACTGAACA -3', containing the *Hind*III and *Xho*I restriction sites (underlined), respectively. The primers were synthesized by *TaKaRa *corporation. The PCR products, which were amplified by high fidelity Taq enzyme (*TaKaRa *Ex TaqTM), were purified by using a TIANprep Mini Plasmid Kit. The purified PCR products were cloned into pMD18-T plasmid (Fig. 7A), transformed into E. coli DH5α competent cells, and the positive recombinant clone was selected by the Amp/IPTG/X-Gal agar plate. Construction of the cloning plasmid was identified by colony PCR, digested with restriction enzymes (*Hind*III and *Xho*I) digestion and fractionated in 1% agarose gels.

The *tgK *was also amplified by other pair of primers. Forward primer (P3) 5'-**AAGCTT**ATGGTAGGAAGACATTGGTG-3' and the reverse primer (P4) 5'-**CTCGAG**AGTTGTCTTATGTCGTACTGAC-3', containing the *Hind*III and *Xho*I restriction sites (underlined), respectively. Cloning procedures and reagents for the *tgK *were the same as these for the *fgK*. Further confirmation of both plasmids pMD18-T/*fgK *and pMD18-T/*tgK *were performed by sequencing.

### Construction of the expression plasmid

The subcloning strategy for constructing the expression plasmid was that the cloning plasmid pMD18-T/*fgK *and plasmid pET-32b(+) (Novagen) were digested with *Hind*III and *Xho*I, and then ligated with DNA ligation kit 2.0 to yield the recombinant prokaryotic expression plasmid pET-32b(+)/*fgK *(Fig. 7B). The recombinant plasmid pET-32b(+)/*fgK *was transformed into competent E. coli DH5α cells. Positive clones were first identified by PCR and then reconfirmed by restriction enzyme digestion.

Also, construct the pET-32b(+)/*tgK *expression plasmid was the same as procedures of the pET-32b(+)/*fgK *expression plasmid. Identical methods were used to indentify the pET-32b(+)/*tgK *expression plasmid. Further confirmation of both subcloning plasmids pET-32b(+)/*fgK *and pET-32b(+)/*tgK *were performed by sequencing.

### Expression of the gK and optimization of expression conditions (temperature, IPTG final concentration and induced durations)

A single positive bacterial colony was inoculated in 5 ml LB broth with ampicillin (100 μg/ml) and allowed to grow overnight at 37°C shaker. The overnight culture was diluted (1:100) in fresh LB broth with ampicillin (100 μg/ml) and grew at 37°C until the OD_600_ value was reached at 0.5-0.6. And then cultures of different expression host bacteria (BL21, Plys, Rosetta) possessing pET-32b(+)/*fgK *or pET-32b(+)/*tgK *expression plasmid grew in LB broth with ampicillin (100 μg/ml) and the expression was induced by 0.2 mM isopropyl-β-D-thiogalactopyranoside (IPTG purchased from Novagen). Bacterial culture was incubated for 4 h with vigorous shaking at 37°C and harvested by centrifugation at 8,000 g for 10 min at 4°C. The pellet was suspended in 10 ml 20×mM Tris-HCl buffer with 0.1 mg/ml lysozyme freezing overnight and then lysed by sonication in an ice water bath. The supernatant and pellet from the induced culture with expression plasmid were analyzed by SDS-PAGE. Also, three kinds of expression host bacteria (BL21, Plys, Rosetta) were used to express this protein, in order to chose the optimal expression host bacteria through analyzing by SDS-PAGE.

Three expression conditions including different temperatures, induced durations and IPTG final concentrations were optimized in order to abundantly express gK and the truncated gK. Bacterial growth conditions were similar to that described above. For optimizing temperature, the bacterial cultures were induced with 0.2 mM IPTG and allowed to grow 4 hours at three different temperatures (25, 30 and 37°C). For IPTG dose optimization, the bacterial cultures were induced with different final concentrations (0.2, 0.3, 0.4, 0.5, 0.6, 0.7, 0.8 and 1.0 mM) and allowed to grow 4 hours at 37°C. For time optimization, the bacterial cultures were induced with 0.2 mM IPTG and allowed to grow for 2.0 h, 4.0 h, 8.0 h and 16.0 h at 37°C. Total tropina harvested from each test was analyzed by SDS-PAGE.

### Purification of inclusion bodies (IB)

In brief, expression host bacteria transformed with expression plasmid, cultured at 37°C in 2L LB medium supplemented with ampicillin (100 μg/ml). The transformed bacteria were induced with 0.2 mM IPTG for 4.0 h. After centrifugation of the bacteria at 8,000 g at 4°C for 10 min, the pellet was suspended in 80 ml 20×mM Tris-HCl buffer with 0.1 mg/ml lysozyme freezing overnight and then lysed by sonication in an ice water bath. The suspension was centrifuged at 10,000 g for 10 min at 4°C, and the pellet was then kept on ice. The pellet was resuspended in 20 ml buffer (4 M urea, 50 mM pH8.0 Tris buffer, 1 mM EDTA, 150 mM NaCl and 0.1% Triton X-100), repeated scrubbing 5 times (every time continuing 10 minutes). After these procedures and centrifugation, the pellet was dissolved in 8 M urea. Then the 40 ul sample was suspended with 10 ul 10×SDS loading buffer, and the mixture was boiled for 10 minutes. After centrifugation, 10 μl sample was taken and analyzed by SDS-PAGE.

### IB solubilization and purification of the truncated gK by IMAC

The IB were initially purified using the methods described above, and the pellet was solubilized in 8 M urea at room temperature (25°C) with gentle shaking for 30 min. The solubilised mixture was then centrifuged at 15,000 g for 10 min, and the supernatant was submitted to further purification. The recombinant His-tagged, truncated gK was purified from the supernatant by immobilized metal affinity chromatography (IMAC) on Ni^2+^-NTA affinity resin following the manufacturer's instruction.

A 20 ml capacity glass column was packed with Ni^2+^-NTA resin matrix (Qiagen GmbH). The column was equilibrated with 4 bed volumes of IMAC buffer (20 mM pH 8.0 Tris-HCl, 500 mM NaCl, 0.5 mM PMSF, and 10 mM imidazole). The supernatant was loaded into the Ni^2+^-NTA agarose resin column pre-equilibrated with IMAC buffer. The column was washed successively with 3 bed volumes of IMAC buffer and 5 bed volumes of IMAC buffer containing 20 mM imidazole. The fusion protein was eluted with IMAC buffer containing 8 M urea and 100 mM imidazole at the flow rate of 1.0 ml/min. The fractions were harvested and analyzed by SDS-PAGE to identify the fusion protein. Also the purified recombinant protein was storaged at -20°C for use.

### Western blots assay

Western blots was performed according to standard procedures [26,34,35]. Protein samples were separated by SDS-PAGE with 12% gel and then electroblotted onto polyvinylidene fluoride (PVDF) membrane. The PVDF membranes were then blocked overnight at 4°C with 10% skimmed milk in TBST (Tris-buffered saline with 0.1% Tween-20, pH 8.0). The membranes were washed and then incubated with rabbit anti-DEV polyclonal antibody while using the normal rabbit blood serum as negative control. The membranes were then washed and incubated with horseradish peroxidase-conjugated goat anti-rabbit IgG (Invitrogen) at 1:5000 of dilution in TBST buffer containing 0.5% BSA. After further washing, immunoreactive protein were visualized by using diamino benzidine (DAB).

### Indirect ELISA

Flat bottomed 96-well plates were coated for 1 h at 37°C with 100 μl per well of truncated gK at the concentrations (2, 3, 4, 5, 6, 7, 8 and 10 μg/ml) in 50 mM carbonate/bicarbonate buffer pH 9.6 and then coated overnight at 4°C. After this procedure, plates were washed three times in PBST (PBS buffer with 0.1% Tween-20) for 5 min each and blocked with 110 ul per well of PBST with 1% BSA for 1 h at 37°C. The sample of the duck anti-DEV positive serum was diluted with 10 gradients ranging from 1:10 to 1:5120 and incubated for 1 h at 37°C. After incubating antiserum, plates were washed and incubated with horseradish peroxidase-conjugated goat anti-duck IgG (Invitrogen) at working concentration 1:500 for 1 h at 37°C. After washing 3 times, 100 μl TMB (3,3',5,5'-tetramethyl-benzidine) was added to the plates followed by exposure for 10 minutes. The reaction was terminated with 2 M H_2_SO_4_ and the OD_450_ value was then read with Elx800 Universal Microplate Reader (Bio-Tek Instruments, Inc., Winooski, VT, USA). In order to accurately determine the optimal dilutions of the truncated gK and serum, the experiment was repeated one time in the same conditions and different time.

To determine the cutoff value for the indirect ELISA, thirty-two negative sera samples from the duck were used in the indirect ELISA to evaluate the cutoff value, which was calculated using the formula: mean of the negative sera values plus three standard deviations (SD) [36]. Each sample was repeated in triplicate wells and the mean value was calculated.

Seven kinds of positive sera samples of the duck anti-DEV, duck anti-DHV, duck anti-AI, duck anti-DVSHD (Duck viral swollenhead haemorrhagic disease) [37], duck anti-R.A., duck anti-E.coli, duck anti-S.E. and one control negative duck serum were used to analyze specificity and sensitivity of the indirect ELISA. These sera samples were prepared by our laboratory. Each sample was repeated in triplicate wells and the mean value was calculated.

## Competing interests

The authors declare that they have no competing interests.

## Authors' contributions

SCZ, GPM and JX carried out most of the experiments and drafted the manuscript. ACC, MSW, DKZ, RYJ, QHL, ZLC and XYC helped in experiments and drafted the manuscript. All authors read and approved the final manuscript.
